# Emotional information-processing correlates of positive mental health in adolescence: a network analysis approach

**DOI:** 10.1080/02699931.2021.1915752

**Published:** 2021-04-22

**Authors:** Sam Parsons, Annabel Songco, Charlotte Booth, Elaine Fox

**Affiliations:** Department of Experimental Psychology, University of Oxford, Oxford, U.K.

**Keywords:** Combined cognitive bias hypothesis, network analysis, adolescent, positive mental health

## Abstract

The combined cognitive bias hypothesis proposes that emotional information-processing biases may conjointly influence mental health. Yet, little is known about the interrelationships amongst cognitive biases, particularly in adolescence. We used data from the CogBIAS longitudinal study (Booth et al., 2017), including 450 adolescents who completed measures of interpretation bias, memory bias, and a validated measure of general mental health in a typically developing population. We used a moderated network modelling approach to examine positive mental health-related moderation of the cognitive bias network. We found that mental health was directly associated with positive and negative memory biases, and positive interpretation biases, but not negative interpretation biases. Further, we observed some mental health-related moderation of the network structure. Network connectivity decreased with higher positive mental health scores. Network approaches allow us to model complex relationships amongst cognitive biases and develop novel hypotheses for future research.

## 1 Introduction

Automatic tendencies to selectively process negative, relative to benign or positive material and environmental events, have been associated with anxiety and depression (for reviews, see Cisler & Koster, 2010; Gotlib & Joormann, 2010; Mathews & MacLeod, 1994, 2005). These biases have been explored in attention, interpretation of ambiguity, as well as in memory. Typically, studies examining selective processing biases in relation to emotional vulnerability have tended to examine these processes in isolation, with few studies examining more than one bias in a single study (Everaert et al., 2012; Hirsch et al., 2006). The combined cognitive bias hypothesis (CCBH: Hirsch et al., 2006), however, proposes that cognitive biases are unlikely to work in isolation, but rather, they influence each other and interact to influence other variables, including emotional vulnerability. For example, increased attention towards negative stimuli may influence how negatively information is interpreted, which would then influence memory for that stimulus. Such a series of causally related negative biases would be expected to propagate emotional vulnerability. In this way, a greater influence of one bias on another may further help to perpetrate a negative cycle of maladaptive cognitive processing related to emotional dysfunction.

The CCBH has been tested with tasks designed to capture the direct effect of one bias on another. For instance, one study (Everaert et al., 2014) used an eye-tracking modification of a scrambled sentences task (Wenzlaff & Bates, 1998). Gaze fixation time on negative words was used to index attention bias, the ratio of negative to total unscrambled sentences was used to index interpretation bias, and a free recall task was used to index memory bias. Two path models were tested, the first omitted relationships between each of the biases, and the second included paths between the biases. The second model provided a better fit to the data, supporting the hypothesis that attention bias influenced interpretation bias, which in turn influenced memory bias. Another study in adults investigated the functional relationships among cognitive biases in a subclinical depressed sample and found that while attentional bias was not directly associated with memory bias, there was an indirect association via interpretation bias (Everaert et al., 2013). Thus, the results supported the CCBH, showing that cognitive biases across different domains do not act in isolation. A further study, conducted in adults, concluded that memory bias is likely to be more effectively modified by targeting emotional processing in another domain, such as interpretation bias (Hertel & Mathews, 2011; Vrijsen et al., 2014). These studies provide support for the CCBH in adults, however, there has been limited research conducted in adolescents.

To our knowledge, only two studies have examined the CCBH in adolescent samples (Klein et al., 2017; Orchard & Reynolds, 2018). Klein et al. (2017) used three cognitive bias measures; an emotional visual search task, a dot-probe task, and an interpretation recognition task to investigate attention and interpretation biases in a normative sample of adolescents. Each cognitive bias predicted a unique variance in anxiety and depression, separately, supporting the CCBH proposition that cognitive biases in different domains contribute separately to emotional vulnerability. A study by Orchard and Reynolds (2018) extended this by showing that the combination of cognitive biases, interpretation bias and negative self-evaluation bias, predicted depression severity more strongly than individual biases alone in both a healthy and clinical sample of depressed adolescents. Both studies suggest that further exploration of adolescent mental health from a CCBH perspective is likely to be highly informative. Adolescence is a developmental period that entails significant cognitive, social, and physiological changes (Fuhrmann et al., 2015; Steinberg, 2015). Therefore, a better understanding of the interrelationships between cognitive biases and their impact on mental health would provide greater insights into how these processes may work in combination to influence psychopathology (Lau & Waters, 2017; Platt et al., 2017; Songco et al., 2020).

### 1.1 Psychological network approaches

The CCBH has a striking overlap with a psychological network approach to emotional disorders. A network perspective on psychopathology views emotional disorders, such as anxiety and depression, as a system of interacting symptoms (Fried, 2017; Fried et al., 2017; Fried & Cramer, 2017). As such, rather than individual symptoms acting alone to influence a disorder, the interrelations among them also play a key role. Borsboom and colleagues have been the driving force behind initiating network analyses in clinical psychology (Borsboom et al., 2011; Schmittmann et al., 2013) and this has resulted in the application of network analysis approaches to psychopathology (Bernstein et al., 2017; Borsboom & Cramer, 2013; Heeren & McNally, 2016; McNally et al., 2015). A common aim of network approaches is to identify plausible, and potentially causal, connections amongst individual symptoms of a disorder (e.g. McNally et al., 2015).

A network theory of mental disorders has been proposed (Borsboom, 2017), including several core principles particularly relevant to the CCBH. These principles are comparable to the, albeit less formal, core elements of the CCBH; namely that we would expect different biases to interact with one another to influence emotional vulnerability and that biases may reinforce one another reciprocally to influence emotional vulnerability. The similarities are such that theoretically applying the principles of the network approach may prove fruitful and provide a more formal and systematic approach to investigate the CCBH. The network approach has been applied primarily to examinations of emotional vulnerability. Though, recent theoretical work has built on this to propose network approaches to resilience research (Kalisch et al., 2019), highlighting the opportunity network approaches offer to examine positive mental wellbeing, as we do in this study. We propose that cognitive biases directly and indirectly influence one another (Borsboom’s direct causal connections principle) and that these interactions among different biases are likely to influence emotional vulnerability as well as emotional wellbeing (Borsboom’s complexity principle).

Network analysis enables the quantification and visualisation of the multivariate dependencies that exist in the dataset. In a psychological network, nodes that represent observed psychological variables (e.g. psychometric tests or indices of cognitive bias) are connected by edges, which represent the observed statistical relationship between them. Regularised partial correlations are often used, thus each edge represents the partial correlation coefficient between two variables after conditioning on all other variables in the network, also known as conditional (in)dependence associations (Epskamp & Fried, 2018). The edge colour is a useful indication of the direction of the association; here positive associations are presented in blue and negative associations in red. The edge weight is used to indicate the strength of a relationship; stronger relationships are represented with thicker edges, whereas weaker relationships are denoted with thinner less saturated edges. Edges with a weight of exactly zero are omitted from the network, indicating that the two variables are conditionally independent (i.e. independent after controlling for all other variables in the network). Regularisation is often used to shrink partial correlation coefficients in order to remove very small associations from the overall network (Epskamp & Fried, 2018), but note that other approaches may be more appropriate depending on the data (Williams et al., 2019). While partial correlation networks can provide insight into predictive mediation (e.g. X predicts Y indirectly via Z) and can suggest potential causal pathways, we must be cautious to not over-interpret causality. In this paper, we use a moderated network analysis to investigate mental health-related changes in the structure of cognitive bias networks.

Network analyses have been used previously to investigate behavioural measures of cognition and behaviour, going beyond self-report measures (Bernstein et al., 2017; Heeren & McNally, 2016). For instance, the interplay between social anxiety symptoms, attentional bias, and attentional control was investigated by Heeren and McNally (2016). Their analysis indicated that the orienting of attention was strongly linked to self-reported fear of social situations, which in turn was strongly related to avoidance of those situations. This has a potentially important clinical implication in that it suggests that interventions targeting attention orientation would positively influence other processes and propagate those benefits throughout the psychological network, resulting in therapeutic benefits (McNally et al., 2015). Similarly, Bernstein et al. (2017) investigated components of executive control and rumination. Their analysis suggested that self-criticism was central to the network with strong down-stream effects on negativity and brooding. Thus, reducing self-criticism may have a wide-reaching beneficial effect on other components of rumination, and represents a potentially useful therapeutic target. Here, it is important to consider that drawing causal conclusions from cross-sectional networks is complicated, perhaps especially for trying to posit targets for intervention (Bringmann et al., 2019). Network analysis approaches provide an informative perspective on the interplay between cognitive processes and components of psychopathology and may help to inform the development of novel clinical interventions.

### 1.2 Cognitive bias approaches to positive mental health and resilience

While information-processing approaches have been widely used to investigate the cognitive mechanisms of emotion dysfunction (for reviews, Gotlib & Joormann, 2010; Lau & Waters, 2017; Mathews & MacLeod, 2005; Yiend, 2010) relatively little research has examined the role of selective information-processing in positive mental health in adults (Carl et al., 2013; Parsons et al., 2016) and even less in adolescents. Positive mental health and mental illness are considered to represent two distinct, albeit inversely correlated, continua (Keyes, 2002, 2005). Low mental health has been found to have additive adverse effects on an individual’s functioning in life, including academic impairment and suicidal ideation (Keyes et al., 2012), as well as mortality (Keyes & Simoes, 2012). An implication of the dual continua model is that positive mental health may be characterised by distinct patterns of selective processing styles or biases, just as the “symptoms” of mental health and mental illness differ from each other. We therefore used Keyes’ mental health continuum (MHC, Keyes, 2009) scale which intends to index psychological, social, and cognitive wellbeing as positive mental health.

Some research has examined factors related to positive mental health within a cognitive-experimental framework – as with the CCBH the majority of this research has been conducted in adults. In the current study, we employed a psychological network analysis approach to investigate the CCBH for positive mental health in adolescents. We use data from Wave 1 of the CogBIAS longitudinal study (Booth et al., 2017; Booth et al., 2019), one of the few studies to collect data for multiple cognitive biases in an adolescent sample (for example, Klein et al., 2017; Orchard & Reynolds, 2018) across different time points. In this study, we focussed on interpretation, and memory biases, to limit the scope to the processes previously implicated in the CCBH (Hirsch et al., 2006). We had intended to include attention bias, but due to poor reliability (see Booth et al., 2019) we opted to omit attention bias from the network analyses.

Our primary aim in this paper is to explore differences in the structure of cognitive bias networks between young adolescents who report higher and lower levels of mental health. We therefore use a moderated network approach, which allowed us to examine the moderating effect of mental health on the relationships between other biases. We note that moderation analyses are often used to search for evidence of causality. However, due to the cross-sectional data, we analyse here we are not aiming to establish causal relations among biases from the network structure. To our knowledge, this is the first study to use network analyses to examine the role that connections in selective processing of emotional information play in positive mental health in an adolescent sample.

## 2 Methods

The data analysed and presented in this paper are drawn from Wave 1 of the CogBIAS longitudinal study (Booth et al., 2017; Booth et al., 2019), which recruited 504 secondary school adolescents (*M* age = 13.4, SD = 0.7) in the UK. Adolescents completed a series of cognitive bias measures (including attention, interpretation, and memory) across three waves of testing. Wave 1 of the CogBIAS study presents an ideal opportunity to examine the CCBH as it applies to early adolescents, specifically with respect to the role these cognitive biases play in positive mental health. To this aim, we use data from wave 1 of the CogBIAS study.^1^

### 2.1 Participants

We first excluded all participants without complete data in all the measures described below, from the original sample of 504 adolescents. This resulted in a final sample of 450 adolescents (*M* age = 13.37, SD = 0.75, 248 female, 75% Caucasian). We used the average score of parent’s highest level of education as an indirect measure of socio-economic status, the median score was 4 (1 = “secondary school,” 2 = “vocational/technical school,” 3=“some college,” 4 = “bachelor’s degree,” 5 = “master’s degree”, 6 = “doctoral degree”).

### 2.2 Procedure

Participants were tested in groups ranging between 13 and 50 students in computer labs either in their own school or in the university. Testing consisted of two, one-hour sessions which were either back-to-back or on different days, depending on school and testing space availability. In each session, participants completed three cognitive tasks, in the same order, followed by a battery of questionnaires (see Booth et al., 2017, for further information on measures not analysed in this paper). With respect to the measures used in this analysis: The interpretation bias task was completed in session 1, while the memory bias task and mental health questionnaire were completed in session 2. Participants were asked to complete both sessions under exam-like conditions, i.e. not talking or looking at their peers’ computer screens. At least two researchers were present throughout the testing sessions to answer any questions and ensure adequate testing conditions were maintained. Ethical approval for this study was given by the National Research Ethics Service (NREC; REC reference: 14/SC/0128; IRAS project ID: 141833).

### 2.3 Measures

As we were interested in differences in network structure of cognitive biases in adolescents reporting high and low positive mental health, we analyse only a subset of the measures included in the CogBIAS study. The CCBH typically describes the relationship between attention, interpretation, and memory biases. We therefore analysed data only from tasks targeting these cognitive processes. We present a brief description of these measures below, however, a complete description of the sample, methods, and design used in the study can be found in a protocol paper for the CogBIAS study (Booth et al., 2017).

#### 2.3.1 Mental health

The mental health continuum-short form (MHC-SF, Keyes, 2009) contains 14 items that index emotional, psychological, and social wellbeing, in order to create a composite measure of positive mental health. Participants are asked to rate how often they have experienced each of the items in the past month, on a 6-point Likert scale from “never” to “every day.” The MHC-SF has shown high internal consistency and discriminant validity (Keyes, 2009; Lamers et al., 2011); in the current sample reliability was high (MacDonald’s Omega ω = 0.95, Cronbach’s alpha α = 0.94, 95% CI [0.93, 0.95]). As our focus was on the network of cognitive biases, and because we examined moderation of the network structure by mental health, we used the total score of the MHC in the networks, rather than the individual items.

#### 2.3.2 Attention bias

An emotional face (angry, happy, and pain) dot-probe task was used to index attention bias (MacLeod et al., 1986) with stimuli from the STOIC faces database (Roy et al., 2009). As with other papers using data from the CogBIAS study, we have opted to omit the attention bias data from our analyses – we also note that reliability issues with the Dot-Probe task have been reported for some time (Schmukle, 2005; Staugaard, 2009). The internal consistency reliability of the attention bias indices (*n* = 448 following removal of two participants for <70% accuracy – we note that these estimates do not reflect the exact sample used in the main analyses due to this) was estimated using the R package *splithalf* (Parsons, 2020) and was below any acceptable threshold, in this sample; angry = 0.02, 95% CI [−0.14, 0.18]; happy = 0.17, 95% CI [0.02, 0.30]; pain = −0.07, 95% CI [−0.22, 0.09]. These outcome measures are unsuitable for any analyses based on correlational measures and were omitted from any further analyses. For full details about the task, see Booth et al. (2019, 2017).

#### 2.3.3 Interpretation bias

The adolescent interpretation and belief questionnaire (AIBQ, Miers et al., 2008) contains ten hypothetical scenarios, five of which are socially oriented and five are non-socially oriented, that are intended to reflect events that are likely to be experienced by adolescents. Participants read the scenario and are presented with a question that addresses a point of ambiguity in the scenario. A positive, a neutral, and a negative interpretation of the scenario are presented and participants rate how likely that interpretation would pop into their mind on a 5-point Likert scale. Participants then choose which interpretation of the scenario they believe to be the most correct. Scenarios are presented in a pseudo-random order. Bias scores were computed by calculating the mean likelihood ratings for positive and negative interpretations of social and non-social situations separately, resulting in four bias indices – social positive; non-social positive; social negative; non-social negative. The reliabilities of each of the bias indices were as follows; social positive (ω = 0.63; α = 0.55, 95% CI [0.48, 0.60]); social negative (ω = 0.81; α = 0.79, 95% CI [0.76, 0.81]); non-social positive (ω = 0.48; α = 0.43, 95% CI [0.36, 0.50]); non-social negative (ω = 0.64; α = 0.58, 95% CI [0.53, 0.64]).

#### 2.3.4 Memory bias

In the self-referential encoding task (SRET), participants were presented with 22 positive and 22 negative words in a random order (the word lists were drawn from Hammen & Zupan, 1984). Each word was presented for 200ms before a prompt “Describes me?” was presented on screen, after which participants responded “yes” or “no” using the “Y” and “N” keys on the computer keyboard. After all, words had been presented, a short distraction task was administered consisting of three simple mathematics questions. Finally, in the incidental recall phase, participants were given three minutes to recall and type in as many words as they could remember. Positive and negative memory bias indices were calculated as the number of positive and negative words, respectively, that were endorsed (participants responded that the word described them) and subsequently recalled (Asarnow et al., 2014). Internal consistency reliability estimates were obtained using the R package *splithalf* (Parsons, 2020): negative = 0.62, 95% CI [0.57, 0.68]; and positive = 0.45, 95% CI [0.37, 0.51]

### 2.4 Data analysis

We conducted a moderated network analysis (Haslbeck et al., 2018) using the *mgm* package, treating mental health as a moderating variable in the network. This allowed us to use the entire sample (*n* = 450) and to treat mental health as a continuous variable, rather than dichotomising our sample. Moreover, moderated networks examine moderation of individual edges, providing a nuanced perspective on any moderating effects of mental health. Haslbeck et al. (2018) demonstrated that a moderated network approach outperforms split-sample methods like network comparison test (as we report in the supplemental analyses) and fused graphical lasso models. We also obtained predictability indices for each node in the network. We then resampled the estimated network 1000 times to obtain confidence intervals around the estimated network structure and moderation effects, thus providing vital information on the stability of the networks. The resampling procedure also allowed us to extract the proportion of non-zero edges and moderating effects. Finally, we computed and visualised three networks conditioned on mental health to illustrate changes in network structure at different levels of mental health.

In an earlier complementary analysis, we compared the network of cognitive biases for a high mental health and low mental health subsample using *NetworkComparisonTest* (van Borkulo et al., 2016), following a tertile split. To avoid redundancy and because these analyses converge on similar conclusions, we present only the moderated network analysis in this paper. Full details and results of the network comparison analysis can be found in the supplemental materials.

## 3 Results

Table 1 presents the descriptive statistics and correlation matrix for all variables included in the moderated network analysis. Supplementary Table 1 presents the descriptive statistics for the high and low groups. Supplementary tables also contain the correlation and covariance matrices for the low MH subsample (S2-3) and the high MH subsamples (S5-6) from the supplemental analyses, and the full sample (S 8-9).

**Table 1.** Descriptive statistics and correlation matrix for the full sample (*n* = 450).

	Mean	SD	(1)	(2)	(3)	(4)	(5)	(6)
MH (1)	40.74	12.59						
IB_S_Pos (2)	2.55	0.62	.37					
IB_N_Pos (3)	3.54	0.64	.33	.41				
IB_S_Neg (4)	3.29	0.88	−.25	−.26	−.17			
IB_N_Neg (5)	3.17	0.72	−.15	−.00	−.19	.51		
MB_Pos (6)	6.84	2.87	.40	.30	.21	−.24	−.15	
MB_Neg (7)	2.51	2.34	−.43	−.28	−.26	.42	.24	−.29

Note*.* MH = positive mental health; IB_S_Pos = social positive interpretation bias; IB_S_Neg = social negative interpretation bias; IB_N_Pos = non-social negative interpretation bias; IB_N_Neg = non-social; MB_Pos = positive memory bias; MB_Neg = negative memory bias.

### 3.1 Moderated network analysis.

We used the *mgm* package (Haslbeck et al., 2018) to estimate the moderated network with positive mental health as the moderating variable. We used cross-validation to select the regularisation parameter. Weak edges are shrunk to zero leading to a matrix of regularised coefficients representing conditional dependence relations. For the nodewise regressions, *k* edge weights are obtained for each *k*-order interaction (e.g. 2 for pairwise interactions and 3 for the moderated effects). We used the OR-rule to combine these weights, (which calculates the mean of all *k* parameter estimates) as the default option and because the AND-rule (which calculates the mean of all k parameter estimates if all estimates are non-zero, otherwise setting the parameter to zero) may be too conservative for the 3-way interactions of interest (Haslbeck et al., 2018). We then extracted predictability indices for each variable following Haslbeck and Waldorp (2018). Predictability refers to the proportion of variance explained by all other nodes in the network.

**Figure 1.**
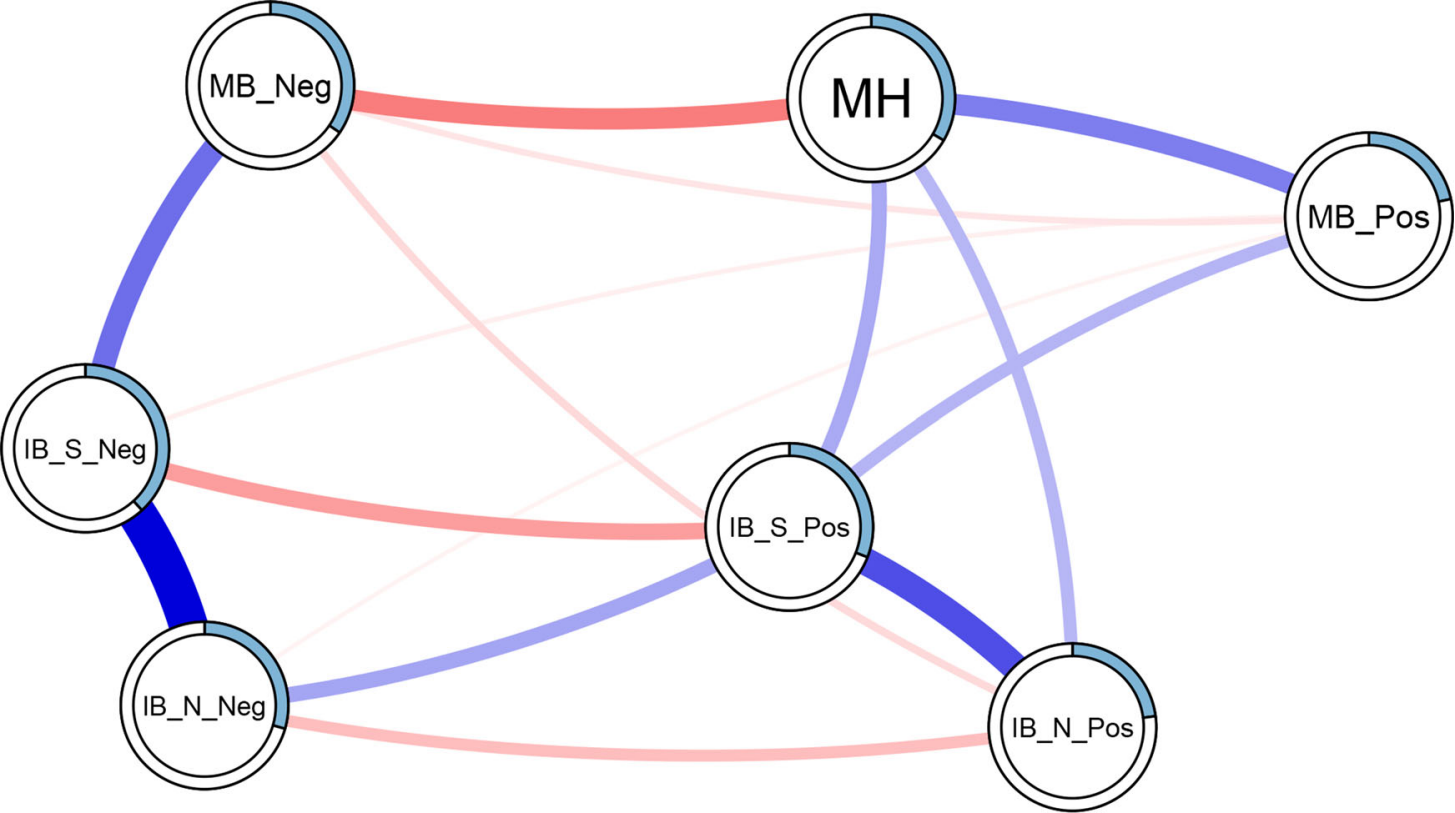
Estimated moderated network of positive mental health, and interpretation and memory cognitive biases. Blue edges represent positive associations, red edges represent negative associations; the width of the edge indicates the strength of this relationship. The shaded area of the pie surrounding each node represents the predictability of that variable, i.e. the variance explained by all other variables in the network. Note that this figure does not visualise the degree of moderation in the networks. Note: MH = positive mental health; IB_S_Pos = social positive interpretation bias; IB_S_Neg = social negative interpretation bias; IB_N_Pos = non-social negative interpretation bias; IB_N_Neg = non-social; MB_Pos = positive memory bias; MB_Neg = negative memory bias.

**Figure 2.**
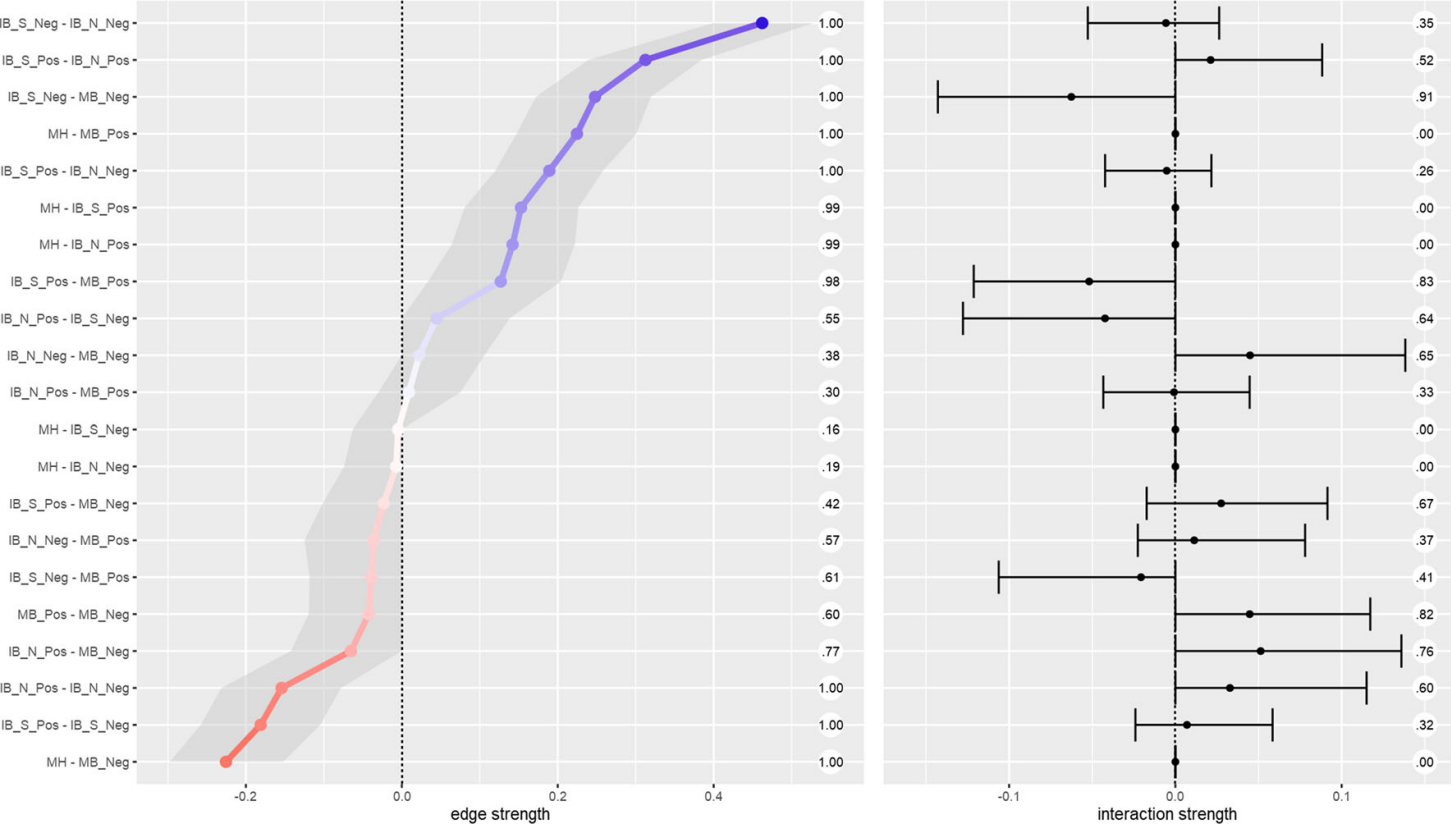
Edge strength and degree of moderation by mental health. The left panel presents the estimated edge weights (these correspond to the network visualisation in Figure 1) from 1000 resamples of the mgm network estimation procedure. The shaded area represents the 95% CI around the estimate. Numbers running down the centre of the figure represent the proportion of non-zero estimates for each edge. The right panel presents the estimated moderating effect of mental health on each edge; the ticks represent the 95% CI around the estimate. The circled numbers represent the number of non-zero moderation effects arising across the resamples. Note: MH = positive mental health. From AIBQ: IB_S_Pos = positive interpretation bias in social scenarios; IB_S_Neg = negative interpretation bias in social scenarios; IB_N_Pos = negative interpretation bias in non-social scenarios; IB_N_Neg = negative interpretation bias in non-social scenarios. From the SRET (endorsed and recalled items): MB_Pos = positive memory bias; MB_Neg = negative memory bias.

The resulting network of pairwise interactions is visualised in Figure 1. For ease of interpretation, we present the moderation effects separately in Figure 2 (a table containing the pairwise and interaction effects for each edge can be found in supplemental Table S10). Mental health was connected most strongly to memory biases – i.e. a negative association with a negative memory and a positive association with a positive memory. For interpretation biases, only edges connecting mental health to positive interpretation biases (social and non-social) were retained. Edges connecting mental health to negative interpretation biases were not retained. Non-social interpretation biases were not directly connected to memory biases (both positive and negative) but were connected via social interpretation biases. The explained variance (predictability metric) of mental health was 33.40%; the predictability of the cognitive biases ranged from 21.70% (positive memory bias) to 38.20% (negative social interpretation bias).

To obtain information about the stability of the moderated network we resampled the moderated network 1000 times. This allowed us to obtain confidence intervals surrounding each individual edge weight. It further allowed us to examine the strength of moderation for each edge and compute confidence intervals around the moderation estimate and the proportion of moderated edges. Figure 2 presents the mean weight and Confidence Intervals for individual edges. Additionally, Figure 2 presents the degree of moderation due to mental health on each edge as a result. Most edges were moderated towards zero with increased positive mental health. Thus, overall we would expect a sparser network at higher mental health, compared to low mental health. The main exception to this trend was the edge connecting social positive and non-social positive interpretation biases. This was the only (likely non-zero) edge to be moderated to be stronger with increases in mental health.

We then ran a parametric bootstrap to further probe the robustness of the moderation effects.^2^ We generated a distribution following a null model, i.e. that there are no moderated edges, and compared this to the point estimates from our full model (Figure 2). We extracted the edge weights matrix from our full model (i.e. Figure 2, left panel) and used the ggmGenerator function from the R package *bootnet* (Epskamp et al., 2017) to simulate 1000 datasets from this model. We applied the full moderated model to each dataset and extracted the edge weights and interaction effects. We then compiled the distributions of interaction effects generated from the null model. We present the null model in the appendices (Table S11 and Figure S12). In most cases, the simulated distributions of interaction effects are near indistinguishable from zero. The point estimate of every interaction effect fell outside the 95% confidence intervals from the null model distributions. This pattern of results gives us more confidence in the moderation effects observed – though the moderation effects are small, and we are careful to avoid over-interpreting the size of results, as discussed below.

### 3.2 Conditioning the network on mental health

**Figure 3.**
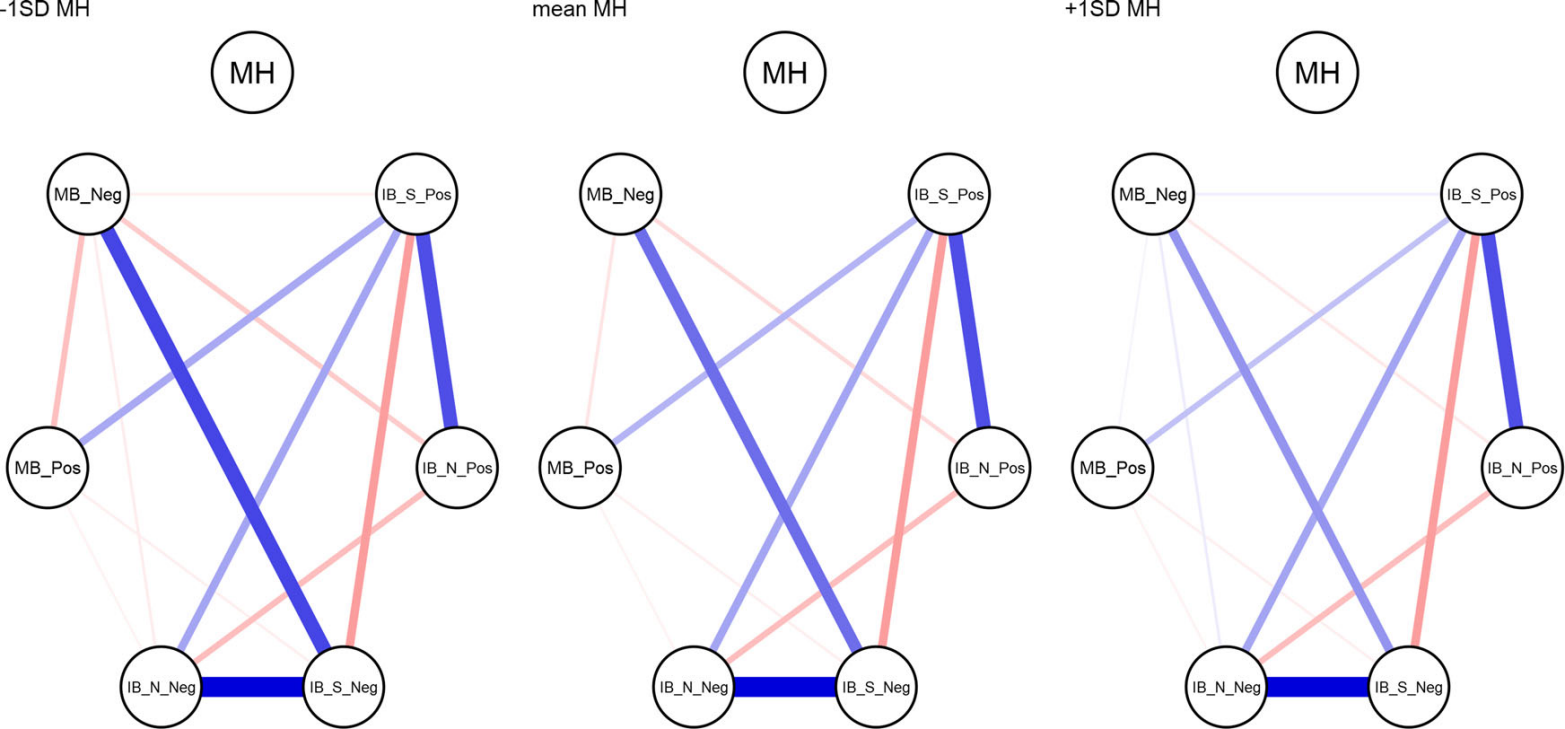
Networks of cognitive biases conditioned on mental health. Mental health is specified at −1SD (left) and +1SD (right) from the mean, and at the mean MH (centre). Conditioning sets mental health to the specified value, and therefore the edges connecting mental health are set to zero (as there is no variance in mental health). Note: MH = positive mental health. From AIBQ: IB_S_Pos = positive interpretation bias in social scenarios; IB_S_Neg = negative interpretation bias in social scenarios; IB_N_Pos = negative interpretation bias in non-social scenarios; IB_N_Neg = negative interpretation bias in non-social scenarios. From the SRET (endorsed and recalled items): MB_Pos = positive memory bias; MB_Neg = negative memory bias.

To highlight the influence of mental health as the moderator of the networks, Figure 3 presents three networks comparing values of mental health. We used the condition() function from the *mgm* package (Haslbeck & Waldorp, 2016) to condition the estimated moderated network (Figure 1) on the mental health moderator. We conditioned the network to −1 standard deviation from the mean, the mean, and +1 standard deviation from the mean on positive mental health (the left, centre, and right panels in Figure 3, respectively).

## 4 Discussion

The present study investigated the CCBH (Everaert et al., 2012; Hirsch et al., 2006) in adolescents using a network approach. We analysed baseline data (at age 12–14 years) from the CogBIAS longitudinal study (Booth et al., 2017; Booth et al., 2019), including interpretation bias and memory bias measures. We excluded attention bias indices from the analyses due to low reliability (we discuss this in the limitations below). We took a moderated network approach treating mental health as a moderating variable in the network of cognitive biases. The results suggest some, albeit small, moderation effects. The edge connecting negative social interpretation bias and negative memory bias was the most moderated edge, both in terms of strength of moderation and in the proportion of resamples that included a non-zero moderation effect estimate. Mental health moderated each edge in 26.50% or more of the resamples (see Figure 2). Three edges were moderated by mental health in more than 80% of the resamples: negative social interpretation bias to negative memory bias; positive social interpretation bias to positive memory bias; and positive memory bias to negative memory bias (Figure 2). Thus, we can have the most confidence that these edges are moderated by mental health, albeit the moderation effects are small. There was a trend for higher mental health to moderate edges towards zero, and therefore, towards a more weakly connected network. This is in line with prior research that observed more densely connected networks of negative affect in a major depressive disorder sample, relative to a healthy control sample (Pe et al., 2015). In other words, higher mental health was associated with reduced associations between cognitive biases.

### 4.1 Implications for the CCBH

The CCBH includes questions of association, causality, and predictive magnitude, with regard to how multiple cognitive biases may contribute to mental health (Everaert et al., 2014). We discuss our results in light of each CCBH question.

Our analyses address the CCBH association question in the broadest sense by showing interconnectedness amongst interpretation and memory biases – we did not analyse the attention bias measures from this sample because of extremely low reliability. Memory biases were most strongly connected to mental health. There were no direct edges connecting mental health and negative interpretation biases, although mental health directly connected to positive interpretation biases. Further, our moderation network model suggests that mental health was related to the network structure itself, in addition to individual biases. We observed some moderation of the network structure by mental health; for example, the edge connecting social negative interpretation bias and negative memory bias were weaker at higher levels of mental health (Figure 2). This supports the CCBH hypothesis that the interrelationships amongst cognitive biases may influence mental health, in addition to any direct relationships between cognitive biases and mental health.

To address CCBH causality questions, we need experimental tasks that integrate multiple cognitive bias processes (e.g. Everaert et al., 2014), or extensive longitudinal research designs. We cannot make inferences from our networks on the causal relations between biases, nor causal relations between the network structure of biases and mental health. Yet, exploratory network analyses are extremely useful for hypothesis generation. One way we can propose causal hypotheses is to examine edges that were not retained in the network. For instance, mental health was not directly connected to negative interpretation biases, when controlling for all other variables in the network. This suggests that negative interpretation biases influence mental health via other biases in the network, e.g. negative memory bias (though, the relationship could also follow the opposite direction, and could also be reciprocal). This follows from the CCBH and suggests a causal chain from negative interpretation bias for social scenarios, via negative memory bias, to influence mental health. Others have argued that it is difficult to modify memory bias directly, and training interpretation bias, with the intent to modify memory biases, may be more effective (Vrijsen et al., 2013). Related to this, our network does not include edges connecting non-social interpretation biases and memory biases.

Network approaches enable us to address the CCBH, albeit via a different lens to traditional approaches. These questions are concerned with whether cognitive biases have additive and/or interactive effects on mental health. For example, previous research has found that combined cognitive biases were more predictive of adolescent depression severity than individual biases alone (Orchard & Reynolds, 2018). Network theory, applied to mental disorders, proposes that highly connected networks are more vulnerable to psychopathology (Borsboom, 2017; also see Kalisch et al., 2019; Pe et al., 2015). Strongly interconnected, causally related symptoms may reinforce one another to propagate a disorder. The network approach conceives of mental health problems as networks of connected symptoms that causally influence each other in a highly dynamic way, as opposed to the traditional view that mental health problems can be classified as distinct clusters of symptoms that are likely to have a single underlying cause. This is a paradigm shift that we have applied to the CCBH, allowing us to move beyond examining only additive and interactive effects of cognitive biases on mental health. Specifically, our moderated network model suggests that greater connectivity amongst biases relates to lower mental health – though we do not aim to make causal claims about these relationships. As we have explored in this paper, network approaches allow us to examine the role of interconnectedness amongst cognitive biases (or symptoms) and relate that structure to mental health.

### 4.2 Limitations

We note several limitations of this study. Larger samples are needed to increase the stability of the moderation effects. Although some edges showed a relatively consistent pattern of moderation by mental health, no edge uniformly showed this moderation across resamples. Though our parametric bootstrap of the null model (no interaction effects) does give us more confidence in the moderation effects observed. Moderation effects are typically small (e.g. Haslbeck et al., 2018), which may explain the lack of stability of some moderation effects. We have endeavoured to interpret the moderation effects with some caution.

We were unable to include attention bias indices in our models. The internal consistencies were so low that we would be unable to make inferences using these measures. We were therefore unable to examine the CCBH as is it usually formulated; including attention, interpretation, and memory bias indices. Although we were unaware of psychometric issues with the dot-probe at the start of this study, it is becoming clear that the task is likely unsuitable for individual differences research (for a summary, see Parsons et al., 2019). In relation to this, our interpretation bias indices did not show optimal reliability. It is possible that this is partly due to the small number of items (5 per bias) that were presented in this task. Moving forward, an important challenge for researchers in this field is to invest more time and resources into developing valid and reliable tasks to assess emotion-related cognitive biases. Otherwise, low reliability will render many CCBH questions unanswerable.

Another important issue is that our network is likely missing important mental health variables. The explained variance of mental health was only 33%. Thus, up to 67% of the variance would be explained by other variables not included in the model, including; other self-reported mental health factors and life events. We opted to include only cognitive bias measures and mental health in our analyses as this was our primary interest of the present paper. We did not include any of the other psychological, social, or cognitive factors thought to comprise positive mental health (Keyes, 2002, 2005). Future work would benefit from selecting measures based on cognitive models of mental health, including symptom-level psychological outcomes. This would provide a more comprehensive test of the CCBH, and we expect would explain much more variance in the model. We also note that explained variance is limited by the reliability of the measures included, further reinforcing the need for greater psychometric scrutiny of cognitive bias measures in general.

Finally, our analyses relate only to the overall relationships amongst cognitive biases, as proposed by the CCBH. With the CogBIAS data, we cannot test direct relationships between biases, in so far as we do not have data from a single task assessing, for example, how attention to stimuli influences the interpretation and subsequent memory for those stimuli (e.g. Everaert et al., 2014). As such, this study cannot address these within-subjects tests of the CCBH. Yet, these results do offer some information on how the interrelations amongst cognitive biases with different stimuli, and valences, differ across levels of positive mental health.

### 4.3 Future directions

Psychological network approaches offer a rapidly developing set of tools for examining the complex interplay amongst symptoms. Indeed, during the initial analysis of this paper, moderated network models were introduced (Haslbeck et al., 2018). This prompted us to reanalyse our data and use the high-low mental health network comparison as a starting point, instead of being our core analysis. Further developments in network approaches offer other important future directions, for example in expected replicability (Williams, 2020), and Bayesian Gaussian graphical models (Williams & Mulder, 2020). Researchers will be able to examine the expected replicability of an exploratory network analyses, such as the one we report here. Moreover, it will be possible to perform confirmatory analyses of networks of cognitive biases via Bayesian hypothesis testing and model comparison. Thus, hypotheses from the CCBH will be directly testable from a network perspective.

Moving forward, we will be able to utilise the full three waves of data in the CogBIAS longitudinal study (Booth et al., 2017) to investigate how these networks change across time. Longitudinal data offers the opportunity to examine the stability of the baseline networks presented in this paper and how they develop throughout adolescence. One study in adults found that symptom networks were related to the longitudinal course of depression; more densely connected networks were associated with persistent major depressive disorder two years later (Van Borkulo et al., 2015). Using a similar approach, we will be able to test whether increased network connectivity at baseline, in early adolescence, predicts consistent levels of negative cognitive biases and poorer mental health in later adolescence. Using all three waves of data, we will also be able to use cross-lagged network models (Epskamp, 2020; Rhemtulla et al., 2019) to model longitudinal changes in the cognitive bias network. We will also be able to examine whether the strength of network connectivity predicts the future network structure. For example, we might hypothesise that a denser network would become denser over time, as biases reinforce one another. In contrast, a sparse network might be expected to remain sparse, as biases remain relatively independent. Longitudinal network approaches offer the opportunity to model complex interactions amongst cognitive biases over time, and therefore, examine the CCBH in greater detail.

To summarise, we applied a moderated network approach to examine the interconnections amongst cognitive biases in a large normative sample of adolescents. To our knowledge, this is the first empirical study to report moderated network models. We have shown the usefulness of a moderated network approach in moving beyond a static symptom network structure, to examine mental health-related changes in the structure. Network analyses offer a valuable tool in examining the CCBH and a novel approach incorporating the complexity of interacting cognitive biases.

## Supplementary Material

Supplementary_Material

## Data Availability

The data and code used for the analyses, and to generate this manuscript can be found online: https://osf.io/mn5ek/ and https://github.com/sdparsons/CogBIAS_W1_networks/

## Notes

1 We used R [Version 3.6.3; R Core Team (2018b)] and the R-packages *bindrcpp* (Müller, 2018), *bootnet* [Version 1.4.3; Epskamp et al. (2017)], *Cairo* [Version 1.5.12.2; Urbanek and Horner (2019)], *corrr* [Version 0.4.3; Ruiz et al. (2019)], *dplyr* [Version 1.0.5; Wickham, François, et al. (2018)], *forcats* [Version 0.5.1; Wickham (2018a)], *foreign* [Version 0.8.75; R Core Team (2018a)], *ggplot2* [Version 3.3.3; Wickham (2016)], *glasso* [Version 1.11; Friedman et al. (2019)], *gridExtra* [Version 2.3; Auguie (2017)], *igraph* [Version 1.2.6; Csardi and Nepusz (2006)], *koRpus* (Michalke, 2018a, 2019), *koRpus.lang.en* (Michalke, 2019), *mgm* [Version 1.2.11; Haslbeck and Waldorp (2016)], *NetworkComparisonTest* [Version 2.2.1; van Borkulo et al. (2016)], *papaja* [Version 0.1.0.9997; Aust and Barth (2018)], *psych* [Version 2.0.12; Revelle (2018)], *purrr* [Version 0.3.4; Henry and Wickham (2019)], *qgraph* [Version 1.6.9; Epskamp et al. (2012)], *RColorBrewer* [Version 1.1.2; Neuwirth (2014)], *readr* [Version 1.4.0; Wickham, Hester, et al. (2018)], *scales* [Version 1.1.1; Wickham (2018b)], *shiny* [Version 1.6.0; Chang et al. (2019)], *splithalf* [Version 0.7.2; Parsons (2020)], *stringr* [Version 1.4.0; Wickham (2018c)], *sylly* (Michalke, 2018b), *tibble* [Version 3.1.0; Müller and Wickham (2019)], *tidyr* [Version 1.1.3; Wickham and Henry (2018)], *tidyverse* [Version 1.3.0; Wickham (2017)], and *wordcountaddin* (Marwick, 2019) for all analyses and figures, and to generate this document

2 We are grateful to Jonas Haslbeck for suggesting this approach
