# Supplementary figures and images for: Emotional information-processing correlates of positive mental health in adolescence: a network analysis approach
Source: Cogn Emot. 2021 Apr 22;35(5):956–69. doi: 10.1080/02699931.2021.1915752 (PMC8372302; doi:10.1080/02699931.2021.1915752)

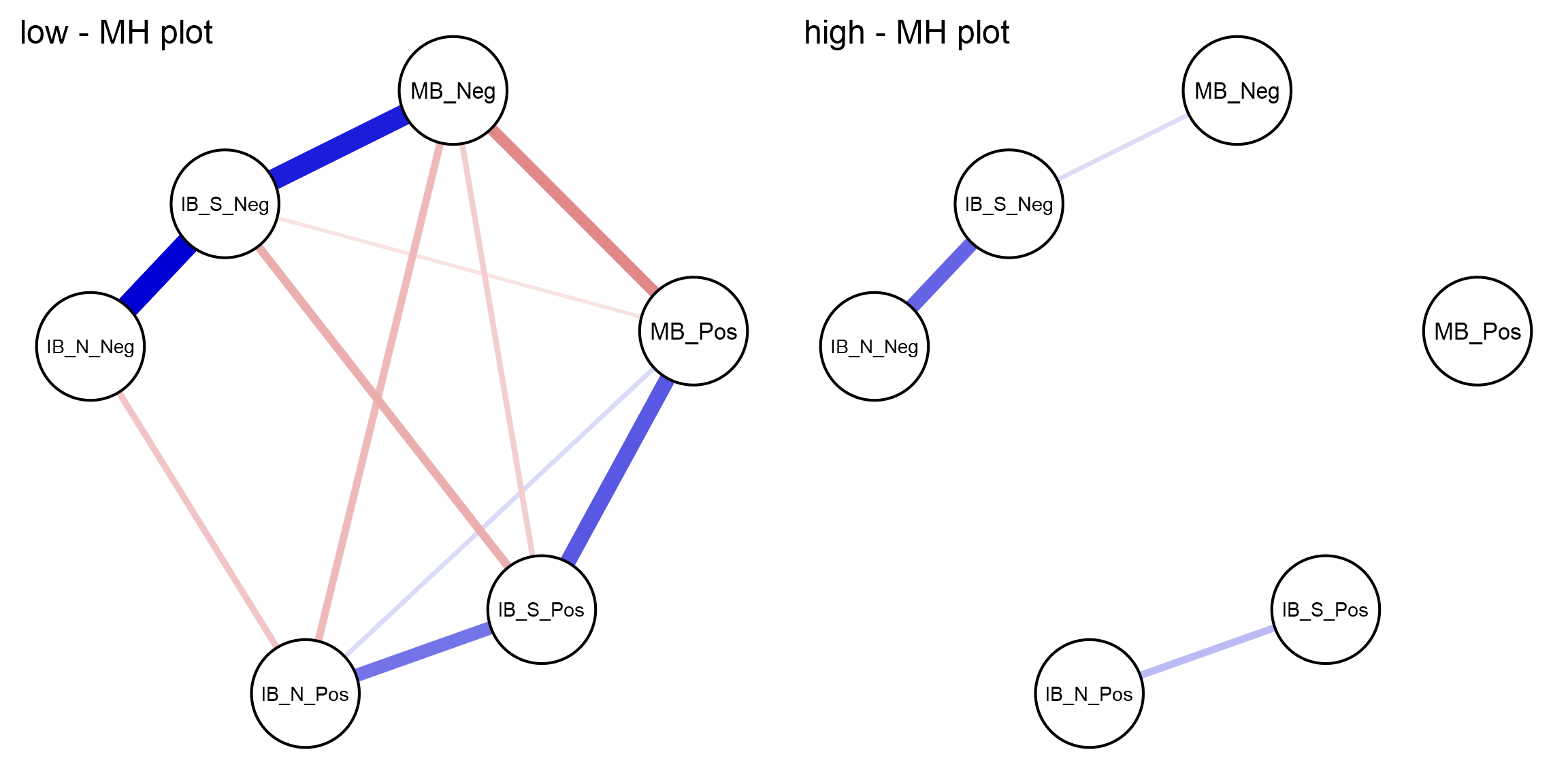

Supplement: Supplementary_Material [file PCEM_A_1915752_SM4712.png]
